# Safety Evaluation of Local Weight-Gain Formulas in the Saudi Arabian Markets

**DOI:** 10.1155/2015/136097

**Published:** 2015-03-05

**Authors:** Nora Abdullah ALFaris, Riyadh Mohammed Al Ashban, Mouna Al Ojayan

**Affiliations:** ^1^Princess Nourah bint Abdulrahman University, Riyadh, Saudi Arabia; ^2^Riyadh College of Dentistry and Pharmacy, Riyadh, Saudi Arabia; ^3^Ministry of Education, Saudi Arabia

## Abstract

*Background*. The utilization of herbal formulas is continuously increasing on the global level. However, assessment of contamination and impurities is the leading challenge in the use of herbal medicines. *Objective*. Assessment of therapeutic application in relation to herbal formulas usage for reducing weight is the objective for this investigation. *Results*. Reduced dietary fibers and fats are common outcomes of herbal usage. 9.8% of the mixtures were contaminated because of lead. However, investigation has indicated <10 microbial counts in herbal products. Increased levels of calcium, minerals, fibers, and lead traces have been identified in the herbals; however, products lacked nicotinamide, riboflavin, and vitamin C. *Conclusion*. It was concluded that majority of herbal products were pure and uncontaminated in order to reduce the complications of obesity efficiently.

## 1. Introduction

The use of herbal medicines is getting increased in the clinical settings within current era. The use of herbal drugs is directly associated with the enhanced wellbeing and wellness of the individuals. The awareness about using herbal medicines is increasing among the professionals for the delivery of quality health care. It is estimated that 80% of the global population is using herbal drugs for addressing health related issues. The increased utilization of the herbal medicines is also developing certain unique types of adulteration and abuse. This health related issue is directly affecting the trust level of the consumers and manufacturers.

## 2. Literature Review

Fatality has been also observed because of herbal drugs related abuse. This issue is considered as the leading challenge for herbal use, which is resulting in unsafe herbal market [1].

This review seeks to enlighten stakeholders in herbal medicine on the need to establish quality parameters for collection, handling, processing, and production of herbal medicine. Moreover, it will also employ such parameters in ensuring the safety of the global herbal market. The processes of good quality assurance and standardization of herbal medicines and products will be also discussed.

Obesity is a serious medical problem, having established links to adverse health outcomes such as hypertension, hyperlipidemia, and insulin resistance. Obesity can be defined as a medical condition, in which an individual suffers from the state of increased body fats. It has been evaluated by various research based articles that condition of obesity will increase the risk of various life threatening medical conditions. The risk of cardiac problems is extremely high due to the condition of obesity. Moreover, uncontrolled state of obesity will also increase the risk of diabetes mellitus [2].

The occurrence of obesity will lead the individuals towards the risk of mortality and morbidity. Similarly, the state of obesity will also increase the risk of hypertension. Hypertension refers to the health issue, in which an individual suffers from increased blood pressure. The state of hypertension will certainly result in the increased risk of renal and cerebral disorders. It is a fact that most of the renal issues and problems are closely associated with the condition of hypertension. Some of the respiratory and metabolic issues are also connected with the state of obesity [3].

As reported by the World Health Organization, at least 2.8 million adults die annually due to obesity [4]. Thus, health care organizations should pay significant attention to this health issue. Recent studies have revealed that a number of overweight and obese people in the Kingdom of Saudi Arabia (KSA) have dramatically increased over the past few decades. Right now, 42.4% of males and 31.8% of females are overweight, whereas 26.4% and 44.0%, respectively, are obese [3, 5].

There are a number of interventions, which are mostly used for addressing the condition of obesity. Various drugs and medicines are used in the clinical settings to eliminate the condition of obesity. At the same time, a number of preventive measures are also used in the social settings to reduce the risk of obesity related disorders [6]. Surgery is also considered as a major strategy, which is only used for extensive cases. It is a fact that proper diagnosis and treatment approaches are necessary to reduce the risk of complications, associated with obesity. Along with these treatment approaches, there are various preventive measures that are used for the control of increased body weight or obesity. A number of research based articles have mentioned that the use of diet and exercises will be significant for the obese individuals to address their problems effectively [7].

A balanced diet and proper exercises are always recommended for controlling body weight. People often turn to herbal products to prevent diseases such as cardiovascular and musculoskeletal ailments and to promote health and wellbeing. A very common example is regarding the use of herbs for stress reduction and weight loss [7, 8]. Nowadays, a trend has developed for the consumption of herbal products. This trend is clearly evident in both developing and developed countries [9]. Rates outlines alternative medicines and some drugs are being marketed as neutral compounds [10]. However, this does not mean that they are free from side effects [11]. Some may even can be contaminated with metals or adulterated with synthetic drugs [9]. Therefore, the contamination in the herbal products will place consumers at higher risks.

Although some alternative therapies may initially reduce weight; however, several have unproven efficacy and may lead to serious problems [12]. Green tea is an example of a product marketed for weight loss without supposed harmful side effects. However, some of the other studies have also shown the existence of side effects, indeed. A study has investigated the impact of green tea on body weight, biochemistry, and hormone profiles of 34 obese Chinese women [13]. They found that although women lose their weight as compared to the control group; green tea led to a significant increase in their triglyceride level [14].

In a systematic review on the use of herbs for weight loss, Pitter et al. [15] concluded that although some herbs were effective for weight loss if linked to dietary changes. However, many had no effect on weight control. Their impact resulted from materials that affect either the volume of food intake or energy balance [15]. A study by Steffen et al. [16] found that 64% of participants had used alternative products to reduce weight with an average monthly expenditure of 33.88 ± 41.1 USD. In addition, 62.3% of participants experience side effects from using of these products. Information about these products was obtained from popular media. Magazines counted for 38.3% of such cases [16].

Alternative medicine usage in KSA is extremely high. In one cross-sectional survey of residents in the Riyadh region, 68% of the participants had used alternative medicines in the previous 12 months [17]. Highlighting the issue of contamination and potential toxicity of herbal products, a study found that eight remedies marketed in the KSA contained synthetic drugs, for example, benzodiazepines and tricyclic antidepressants (in sedative doses) and cyproheptadine [9]. Furthermore, another study has found that the herbal supplements in KSA contained factors that may either enhance or diminish the appetite. Moreover, adverse effects of the contamination will make a direct impact over the nervous system. There is a paucity of data on the use of herbal products marketed in KSA for weight loss. Therefore, the present study aims to evaluate the quality of weight-reducing products marketed in KSA.

## 3. Objective

This investigational project was intended to assess the safety and quality of herbal formulas.

## 4. Materials and Methods

Initially, local vendors were used for the purpose of data collection in relation to famous herbal medicines for weight loss. It has been evaluated by World Health Organization that standardization and quality control processes are strongly connected with physicochemical evaluation. Various different aspects of the drugs and medicines can be easily assessed with the help of crude oil. Selection of crude oils, safety approaches, stability appraisal, absolute handling of crude products, and efficacy methods can be used for the physicochemical evaluation of the formulas. The approach of documentation was also used for fulfilling ethical considerations effectively [18]. Following examinations play a major role in the assessment of herbal drugs and medicines.

### 4.1. Macro- and Microscopic Examination

This approach is mostly used for the appraisal and investigation of formulas in relation to adulterants and abuse [19].

### 4.2. Foreign Organic Matter

Foreign organic matter is also used in the industrial settings for the assessment of drugs. Pure drug or medicine can be easily retrieved with the help of foreign organic matter [20].

### 4.3. Fat, Ash, Carbohydrate, Protein, and Fiber

**Figure 1:**
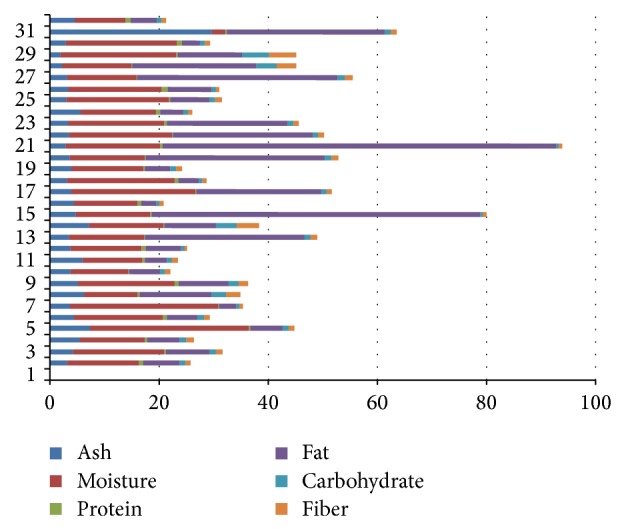


Carbohydrate, fiber, ash, fat, and proteins are usually used for the evaluation of purity in relation to herbal formulas. Suphated ash, acid insoluble ash, total ash, and water soluble ash are common examples (Figure 1) [21].

### 4.4. Moisture Content

Evaluation of moisture content is another very common approach for purity assessment. Assessment of moisture content can easily identify the occurrence of errors in the herbal formulas. Abridged figures of moisture content will specify enhanced constancy of herbal drugs against the process of degradation [22].

### 4.5. Extractive Values

Extractive values are another common approach, which is study for quality assessment. Extractive values refer to analytical weights of component, related to crude oil. Diverse forms of solvent environment are mostly used for extracted components of crude oil [23].

### 4.6. Crude Fiber

Evaluation of quality can be also assessed with the help of crude fiber. Determination of woody components can be done due to crude fiber. Evaluation of purity is also associated with crude fiber [24].

### 4.7. Qualitative Chemical Evaluation

Qualitative chemical evaluation is another very common strategy, which can be used in the professional environment for evaluating quality of drugs. Identification and characterization of the crude drugs can be also achieved because of this approach [22]. This approach encompasses miscellaneous analytical techniques for identifying and isolating active constituents. Phytochemical screening approaches can be also used for the identification of botanic material, purifications of the drugs, extraction of the solvents, and classification of the active components [25]. This strategy can be also used for assessment of herbal drugs effectively.

### 4.8. Chromatographic Examination

Chromatographic examination is another significant approach, which is used for quality assessment. This significant approach involves the identification of drugs by using the effective constituents as markers [22].

### 4.9. Chromatography

Another very common technique is regarding chromatography; it is effectively used for a significant number of reasons in the industrial environment. This approach is defined as a study regarding the distribution of diverse molecules. It is a fact that molecules are usually distributed because of altered structure and composition. Moreover, this strategy is mostly used for the preparation of certain materials and products. The molecules, which are used in the test preparation, will definitely have the close connections with stationary support. Stationary support will be significant for the distribution of similar molecules. Strong association of the molecules with support will move somehow slower than the molecules, having weaker association [26]. A number of molecules are spirited within the support medium during movement. Furthermore, chromatographic distribution required various support media for deriving out positive outcomes. “high performance thin layer chromatography (HPTLC),” “immobilized silica on glass plates,” “volatile gases,” “hydrophilic liquids,” “papers of chromatography,” and “insoluble molecules” are extremely common support materials [27].

“High performance thin layer chromatography (HPTLC)” is a tremendously momentous and significant quality valuation technique, which is used in the professional environment. Assessment and appraisal of the botanical materials can be done with this strategy. Additionally, this technique will also be obliging for investigating compounds in terms of cost effectiveness and efficiency. At the same time, various samples can be also used in one analysis for reducing the time. Similar analysis can be simply done on the basis of various diverse wavelengths of the light [28]. Moreover, HPTLC will also provide a detailed and comprehensive profile of the botanical products. Therefore, HPTLC play a vital role in the quality evaluation of the herbal drugs [27].

Initially, data were collected from local vendors regarding the three most popular herbal products with weight reducing claims. Subsequently, the approximate chemical compositions of the selected herbal products were obtained using calorimetry. All of the guidelines of the Association of Analytical Communities have been followed in the procedure [15]. The content of moisture, ash, protein, fat, mineral, vitamin, and caffeine heavy metals (lead, mercury, arsenic, and cadmium) were measured. Shimadzu AA-6800 Atomic absorption spectrophotometer and Shimadzu Scientific Instruments, 7102 Riverwood Drive, Columbia, MD, USA, were used. Microbiological assays were also conducted in order to assess the total viable aerobic bacterial count weight-reducing medications such as or listat, sibutramine, or steroids using the HPLC system Shimadzu Scientific Instruments, 7102 Riverwood Drive, Columbia, MD, U S A [29].

### 4.10. Statistical Analysis

SPSS v.15 (statistical package for social sciences, version 15, SPSS Inc., Chicago, USA) was used for the purpose of statistical analysis. The study used *t*-test and Chi-square test techniques for the assessment of the values obtained in both the treated and the control groups during the study [30]. *P* values of <0.05 were considered statistically significant.

## 5. Results

### 5.1. Nutritional Content

The nutrient composition of the herbal products is mentioned in Table 1. Protein was the major contributor of energy followed by carbohydrates and fat. More than 46.88% (number 15) herbals have protein more than 15%. More than 90.63% (29) of samples have protein more than 10%. Although the information in the label shows that they are content herbals such as protein, the role of protein in decreasing weight needs to be reevaluated. The fat content of the herbal products was relatively low. HP3 contained the highest level of fiber, followed by HP 2 and HP 1. HP 1 contained the highest mineral content (11.71%) followed by HP 3 (11.05%) and HP 2 (7.73%). HP 2 contained the highest level of calcium (36.19 mg). HP 2 contained traces of lead (23 ppm). However, no other heavy metals were evident in any of the products. Biochemical assays found caffeine was height 21.9% (7) in HP 1 (720 mg) and HP 3 (1203 mg). The herbal products lacked vitamin C, nicotinamide, and riboflavin. HP 3 contained the highest amount of thiamine (53.71 mg) followed by HP 2 (44.76 mg), whereas HP 1 contained minimal amounts (9.91 mg). HP 2 had the highest pyridoxine content (5.13 mg) followed by HP 3 (4.40 mg) and HP 1 (2.16 mg).

### 5.2. Calories, Mineral, Calcium, and Heavy Metals

The fat and fiber contents of the herbal products were relatively low. HP 21 contained the highest level of calorie, followed by HP34 and HP 1. HP 1 contained the highest mineral content (11.71%) followed by HP 3 (11.05%) and HP 2 (7.73%). HP 2 contained the highest level of calcium (36.19 mg). HP 9 contained traces of lead (45 ppm) fallow by HP6 (23 ppm) HP34 (22 ppm). However, no other heavy metals were evident in any of the products. Biochemical assays found caffeine in HP 10 (1760.76 mg), HP 1 (1240.10 mg), HP 1 (720 mg), and HP 3 (1203 mg). The herbal products lacked vitamin C, nicotinamide, and riboflavin. HP3 contained the highest amount of thiamine (53.71 mg) followed by HP 2 (44.76 mg), whereas HP 1 contained minimal amounts (9.91 mg). HP 2 had the highest pyridoxine content (5.13 mg) followed by HP 3 (4.40 mg) and HP 1 (2.16 mg). Contents of vitamin and mineral of herbal mixtures are presented in Table 2.

### 5.3. Contamination of the Herbal Products

The microbiological assays indicated that all the herbal products studied contained less than 10 microbial counts (including total viable fungal count). Evaluation of the microbiology has been mentioned in Table 3. None of the products contained weight-reducing medications such as orlistat, sibutramine, or steroids. This is because all of them do not have any substance to stimulate microbial growth such as peanut and honey; while the heavy metal l assays indicated that samples 6, 9, and 34 have contamination with lead 22, 45, and 23 ppm. Complete information about the impurities and microbial contamination is presented in Table 4.

## 6. Discussion

The use of herbal medicines is continuously increasing in the developing and developed countries. A significant number of research based articles have proved that the use of herbal drugs will be helpful for the patients to address various serious problems. A study was conducted by Kunle et al. [31], which have focused on the use of herbal medicines in the clinical settings. The study has mentioned that herbal medicines have enough capability to cope with diverse range of medical conditions. The study has also shown that the risk of various medical conditions can be easily reduced due to herbal medicines [31].

The current study has shown that the utilization of herbal medicines will be helpful for the health care professionals to address complicated health issues. This statement has been verified by various authenticated articles. A study was conducted by Damery et al. [32], which has focused on the use of herbal medicines against cancer. The study has mentioned that the continuous use of the herbal medications will be significant for the patients to overcome the condition of cancer effectively. The herbal medicines are always made from the botanic products, which will increase the regulation of body processes. Therefore, the problem and issue of overgrowth can be easily addressed with the help of herbal medication [32].

This study has clearly mentioned that the use of herbal medicines will make a positive impact over the condition of obesity. The findings of this study have shown that the use of herbal medicines will be significant for the obese individuals to reduce their weight effectively. Similar findings have been identified in another study, which was conducted by Hasani-Ranjbar et al. [33]. The study has mentioned that the utilization and consumption of the herbal medications will be helpful for the metabolic system to reduce the body weight. It has been mentioned by the study that the state of obesity will make a negative impact over various body systems. The cardiac system and metabolic system will be directly affected by the enhanced condition of obesity. The use of herbal drugs will help the different systems of the human body to become healthy. Moreover, the immune system of the human body will also become improved due to the use of herbal medications. It is a fact that the consumption of herbal medications will certainly result in the improved health status of the obese patients. Additionally, the herbal medications will also affect the digestive system for proper breakdown of the diet [33]. Therefore, the study has clearly supported the findings of this project in a positive way.

Another study was conducted by Najm and Lie [34], which have described the importance of herbal medications for various diseases and health related issues. The study has shown that the use of herbal medications will be significant for the individuals to address the condition of diabetes mellitus and obesity. The study has further elaborated that the consumption of the herbal medicines will be helpful for the health care professionals to reduce the risk of cardiovascular conditions and metabolic issues. The state of diabetes mellitus can be also prevented with the help of herbal medications [34]. Therefore, this study is also supporting the findings of our project.

Alwakeel [35] conducted a comprehensive research based study, which has focused on the contamination of herbal products. The study has mentioned that the trend of using herbal mediations is increasing in the developed and developing countries. The reason behind this statement is that the use of herbal medications is deriving out positive and healthy outcomes in relation with health status of the patients. The study has further described that the use of herbal medications is directly associated with the reduced state of various chronic and acute medical conditions [35]. Therefore, the health care professionals are using herbal medicines for their patients to eliminate a range of medical conditions. This selected study has supported the statements and arguments of our project.

The selected past study has also shown that the condition of drug contamination is a serious threat to the wellbeing and wellness of the individuals. It is a fact that the factor of contamination will be hazardous for the individuals in regards to their health status. It is a fact that excessive use of contaminated herbal medications will certainly result in the deprived health status of the patients. It is also a fact that the use of contaminated herbal medications will increase the risk of mortality and morbidity. Therefore, it is mentioned in this study that the proper use of strategies is necessary for the evaluation and assessment of the product quality.

Our research project has used a significant number of quality measurement techniques for the assessment of herbal products. Li et al. [36] have conducted research based articles, which have focused on the quality assessment of the herbal medications. The study has shown that the use of herbal medications is directly associated with the improved health status of the patients. However, the condition of contamination will affect the general health status of the patients. It is a fact that the use of herbal medications, having contamination, will make a serious impact over the health and body systems of the human body [36]. The study has shown that the pharmaceutical industries and other institutions should use proper techniques and approaches for the assessment of quality in relation to the herbal products.

## 7. Conclusion

The consumption and utilization of the herbal formulas are rising on global level. The chief rationale for using herbal drugs is its noteworthy outcomes. The usage of herbal medicines will be significant for the obese individuals to address their health issues regarding overweight. Standard use of herbal medications will certainly result in the reduced risk of obesity and comorbidities. In addition, quality assessment is extremely necessary for reducing the health related risks, associated with contaminated formulas. Therefore, it can be said that pure herbal formulas can easily reduce the weight of obese individuals effectively.

## Figures and Tables

**Table 1:** Chemical composition of local formulas (g/100 g) on fresh water.

Number	Ash	Moisture	Protein	Fat	Carbohydrate^*^	Fiber	Energy (Kcal/100 g)
1	11.71	3.23	13.12	0.84	6.62	1.02	86.52
2	7.73	4.29	16.65	0.32	7.88	1.29	101.03
3	11.05	5.49	11.93	0.53	5.75	1.36	75.51
4	7.37	7.41	29.05	0.32	5.81	1.12	142.32
5	7.80	4.46	16.26	0.76	5.57	1.15	94.16
6	5.26	3.71	27.12	0.19	3.09	0.65	122.55
7	20.23	6.29	9.75	0.41	13.20	2.61	95.49
8	7.39	5.28	17.58	0.70	9.22	1.76	113.50
9	8.02	3.79	10.52	0.23	5.61	0.92	66.59
10	6.23	6.02	10.95	0.38	4.09	0.97	63.58
11	7.33	3.80	12.97	0.81	6.51	0.53	85.21
12	9.56	3.50	13.76	0.11	29.31	1.12	173.27
13	7.90	7.21	13.55	0.24	9.41	3.92	94.00
14	8.89	4.69	13.61	0.36	60.20	0.51	298.47
15	5.18	4.48	11.51	0.85	2.52	0.75	63.78
16	6.31	3.98	22.74	0.21	22.90	0.88	184.48
17	6.36	3.18	19.59	0.78	3.76	0.69	100.46
18	7.71	4.00	13.07	0.43	4.58	1.09	74.43
19	6.80	3.68	13.76	0.12	32.83	1.25	187.46
20	6.74	2.90	17.31	0.40	72.08	0.57	361.16
21	4.42	3.50	18.81	0.24	25.65	0.99	179.98
22	6.55	3.28	17.64	0.51	22.18	1.00	163.89
23	8.10	5.69	13.70	0.88	4.21	0.77	79.55
24	8.42	3.03	18.79	0.29	7.04	1.15	105.91
25	7.35	3.41	17.10	1.02	8.17	0.66	110.21
26	8.75	3.14	12.70	0.14	36.77	1.40	199.11
27	11.30	2.23	12.68	0.26	22.73	3.60	144.01
28	21.39	1.90	21.25	0.30	11.80	4.92	134.93
29	4.99	2.89	20.34	1.07	3.16	0.94	103.61
30	37.51	29.67	2.43	0.27	29.02	1.10	128.20
31	15.60	4.63	9.25	0.98	4.72	0.87	64.72

Means	25.71	9.68	4.99	15.47	0.48	15.69	1.34
Standard deviation	14.75	6.48	4.77	5.32	0.30	16.84	1.03

^*^Total carbohydrates calculated by difference.

**Table 2:** The Content of vitamin and mineral of herbal mixtures (mg/100 g).

Number	Thiamine	Ascorbic acid	Nicotinamide	Pyridoxine	Riboflavin	Caffeine	Calcium
1	9.91	0	0	2.16	0	720	27.50
2	44.76	0	0	5.13	0	0	36.19
3	53.71	0	0	4.40	0	1203	35.33
4	24.49	0	0	10.10	0	0	30.63
5	68.54	0	0	7.18	0	0	58.00
6	51.97	0	355.34	3.72	0	1760.76	11.19
7	0.94	0	61.40	0.61	6.10	0	49.18
8	28.71	0	0	4.00	3.25	0	23.80
9	48.63	0	0	0.45	6.30	0	22.71
10	3.01	0	3.83	4.81	21.19	0	29.91
11	11.66	0	0.00	11.88	12.18	0	27.53
12	20.20	0	0.00	14.35	15.43	0	36.03
13	5.36	66.83	10.83	37.50	26.12	0	33.02
14	158.55	3.70	62.49	2.94	9.38	275.25	19.80
15	210.61	0.00	38.84	0.00	6.53	401.74	23.13
16	0.00	0.00	0.00	2.02	9.29	967.10	7.66
17	0.00	0.00	9.13	3.07	34.68	698.6	22.79
18	0.00	6.40	0.00	2.38	21.65	0	30.42
19	0.00	11.97	0.00	0.00	3.46	4.60	22.82
20	0.51	3.82	0.82	3.74	14.98	0	28.53
21	7.24	0.22	0.00	2.98	21.13	0	11.28
22	6.21	0.00	21.00	12.22	149.20	1240.10	31.67
23	1.40	0.00	8.55	0.00	18.14	0	23.98
24	0.50	0.00	0.93	0.71	17.11	0	22.41
25	38.12	17.59	31.38	2.47	19.15	0	20.43
26	8.14	1.63	1.37	0.24	25.97	0	23.78
27	3.39	31.38	0.00	0.71	23.63	0	35.24
28	0.00	0.00	0.00	0.00	0.00	0	10.04
29	27.64	0.00	0.00	197.57	8.01	0	7.26
30	22.07	1.47	0.00	0.39	2.11	0	7.84
31	10.22	0.00	0.00	1.14	24.43	656.75	52.76

**Table 3:** Microbiological examination of local formulas in KSA (CFU/g^−1^)^*^.

Number of classification	Total viable count of fungi	Microbial counts
1–31	<10	<10

^*^Colony forming unit.

**Table 4:** Heavy metal of the mixtures (PPM).

Number	Cadmium	Arsenic	Mercury	Lead
1	Nil	Nil	Nil	Nil
2	Nil	Nil	Nil	23
3-4	Nil	Nil	Nil	Nil
5	Nil	Nil	Nil	45
6–19	Nil	Nil	Nil	Nil
20	Nil	Nil	Nil	22
21–31	Nil	Nil	Nil	Nil
